# Revisiting left atrial volumetry by magnetic resonance imaging: the role of atrial shape and 3D angle between left ventricular and left atrial axis

**DOI:** 10.1186/s12880-021-00701-5

**Published:** 2021-11-09

**Authors:** Jouni K. Kuusisto, Pauli A. K. Pöyhönen, Jani Pirinen, Lauri J. Lehmonen, Heli P. Räty, Nicolas Martinez-Majander, Jukka Putaala, Juha Sinisalo, Vesa Järvinen

**Affiliations:** 1 Heart and Lung Center, Helsinki University Hospital and University of Helsinki, Haartmaninkatu 4, PO Box 340, 00029 Helsinki, Finland; 2 HUS Medical Imaging Center, Clinical Physiology and Nuclear Medicine, Helsinki University Hospital and University of Helsinki, Helsinki, Finland; 3 Internal Medicine, HUS Porvoo Hospital Area, Porvoo, Finland; 4 HUS Medical Imaging Center, Radiology, Helsinki University Hospital and University of Helsinki, Helsinki, Finland; 5 Neurology, Helsinki University Hospital and University of Helsinki, Helsinki, Finland

**Keywords:** Left atrium, Volume assessment, Shape, Axis, Cardiac magnetic resonance imaging, Sphericity

## Abstract

**Background:**

Accurate measurement of left atrial (LA) volumes is needed in cardiac diagnostics and the follow up of heart and valvular diseases. Geometrical assumptions with 2D methods for LA volume estimation contribute to volume misestimation. In this study, we test agreement of 3D and 2D methods of LA volume detection and explore contribution of 3D LA axis orientation and LA shape in introducing error in 2D methods by cardiovascular magnetic resonance imaging.

**Methods:**

30 patients with prior first-ever ischemic stroke and no known heart disease, and 30 healthy controls were enrolled (age 18–49) in a substudy of a prospective case–control study. All study subjects underwent cardiac magnetic resonance imaging and were pooled for this methodological study. LA volumes were calculated by biplane area-length method from both conventional long axis (LAV_AL-LV_) and LA long axis-oriented images (LAV_AL-LA_) and were compared to 3D segmented LA volume (LAV_SAX_) to assess accuracy of volume detection. 3D orientation of LA long axis to left ventricular (LV) long axis and to four-chamber plane were determined, and LA 3D sphericity indices were calculated to assess sources of error in LA volume calculation. Shapiro–Wilk test, Bland–Altman analysis, intraclass and Pearson correlation, and Spearman’s rho were used for statistical analysis.

**Results:**

Biases were − 9.9 mL (− 12.5 to − 7.2) for LAV_AL-LV_ and 13.4 (10.0–16.9) for LAV_AL-LA_ [mean difference to LAV_SAX_ (95% confidence interval)]. End-diastolic LA long axis 3D deviation angle to LV long axis was 28.3 ± 6.2° [mean ± SD] and LA long axis 3D rotation angle to four-chamber plane 20.5 ± 18.0°. 3D orientation of LA axis or 3D sphericity were not correlated to error in LA volume calculation.

**Conclusions:**

Calculated LA volume accuracy did not improve by using LA long axis-oriented images for volume calculation in comparison to conventional method. We present novel data on LA axis orientation and a novel metric of LA sphericity and conclude that these measures cannot be utilized to assess error in LA volume calculation.

***Trial registration*:**

Main study Searching for Explanations for Cryptogenic Stroke in the Young: Revealing the Etiology, Triggers, and Outcome (SECRETO; NCT01934725) has been registered previously.

**Supplementary Information:**

The online version contains supplementary material available at 10.1186/s12880-021-00701-5.

## Background

Left atrial (LA) size has many clinical correlations in cardiac disease. In addition to maximum LA volume, minimum volume and LA volume dynamics have been shown to have clinical significance in all-cause mortality, major adverse cardiac events, heart failure, stroke, systemic thromboembolism as well as atrial fibrillation burden [1–7]. Therefore, accurate LA volume measurement is crucial for using this parameter in clinical decision-making and research.

LA size has been estimated with diameter and area measurements, area-based calculations, and direct volume measurements. Direct three-dimensional (3D) volume measurement by cardiovascular magnetic resonance imaging (CMR)—that is defining LA boundaries in a set of parallel of images covering all LA—has been validated with human cadaveric casts [8]. The European Association for Cardiovascular Imaging consensus statement recommends volume assessment over LA area, however by biplane methods rather than direct 3D volume measurements due to availability of reference values, limited access to 3D analysis tools and its time constraints in clinical setting [9].

Methods where volume is calculated from 2D images, which do not cover the whole chamber, introduce error based on geometrical assumptions. LA volume calculation is typically based on left ventricular (LV) two- and four-chamber long axis views (2CH, 4CH), which lie approximately in 60 degrees angle to one another. A source of error is introduced as chamber morphology reflected in three-chamber view (3CH) is not appreciated. Accuracy of such LA volume calculation method and 3D volume measurement were studied in vitro by atrial cadaveric casts with cardiac ultrasound [10]. Typical CMR long axis images are oriented along LV long axis which usually is not parallel with true axis of LA [11]. Images collected in this manner may not be representative of the largest area of LA and contribute to volume misestimation. With echocardiography, atrial focused apical views are recommended in the guidelines to calculate LA volumes, as this improves agreement with 3D measured volumes [12]. Left atrial shape, and specifically its sphericity, has been parameterized by different approaches, for example by calculating the ratio of transverse and longitudinal diameters of LA [13], the ratio of chamber volume to sphere whose diameter is equal to largest length of LA in 2D images [14], or by more advanced computational methods [15, 16].

We aimed to investigate different methods for LA size determination in a study population with no apparent heart disease, who underwent a comprehensive CMR to study possible sources of cardiogenic emboli or served as controls for the study. This material allowed us to perform a methodological study on whether there still exists a rationale to use 2D methods in contrast to volumetric 3D methods. We used three-dimensional methods to define LA and left ventricular axes, LA axis rotation, and LA sphericity. By using these measures, we assessed whether differing LA and left ventricular axes, LA axis orientation or LA shape explain possible differences in 2D calculated volumes in comparison to 3D segmented volumes. We hypothesized that the error in calculated volumes is partly attributed to differing LV and LA axes, and that this error can be mitigated by acquiring specific LA long axis images for LA volume calculation. We also studied if LV systolic function and LA dynamics are reflected in the LA-LV angle or its change during the cardiac cycle.

We have previously published a case–control study by CMR in this same study population, where we showed an association of left ventricular non-compaction and cryptogenic stroke. Baseline data and basic cardiac structure measurements per each group are also reported in this study. [17] No statistically significant differences were found in LV volumes or mass, LV ejection fraction, or left atrial maximum indexed volume (Additional file 1: Table 1).

## Methods

### Materials

Searching for Explanations for Cryptogenic Stroke in the Young: Revealing the Etiology, Triggers, and Outcome (SECRETO; NCT01934725) is an international prospective multicenter case–control study of young adults (age 18–49) presenting with a magnetic resonance imaging verified first-ever ischemic stroke of undetermined etiology. Patients were included after ruling out established causes of ischemic stroke. Standardized protocol included brain magnetic resonance imaging and computed tomography or magnetic resonance imaging of intracranial and extracranial vessels, cardiac imaging with transthoracic and transoesophageal echocardiography with bubble test, transcranial Doppler ultrasound with bubble test, 12-lead ECG and at least 24-h Holter ECG. The main study protocol has been published in more detail previously [18]. In this single-center substudy, 30 healthy controls and 30 patients with a cryptogenic stroke were examined with a comprehensive CMR. Patients and controls were pooled for the purpose of this methodological study. This study complies with the Declaration of Helsinki. Written informed consent was obtained from all study participants and the study was approved by the Ethics Committee of Helsinki and Uusimaa Hospital District.

### CMR protocol

All subjects were imaged using a 1.5 T Avanto^fit^ magnetic resonance imaging system (Siemens Healthcare, Erlangen, Germany). A 32-channel body receiver coil was used in combination with ECG-gating. Gated three-direction localizer was used as a basis for image acquisition. Specific technical parameters for each sequence type used are reported in Additional file 1: Table 2.

Half-Fourier-acquisition single-shot turbo spin echo sequence was first acquired in transaxial planes covering the entire heart. The LV 2CH view was determined in this transaxial set of sections using the built-in three-point plane planning tool, with points in LV apex, and both caudal and cranial center of the mitral ring. Balanced steady-state free precession cine images with cartesian sampling were then acquired in 2CH view, which was used for orienting short-axis (SAX) cine images covering the entire heart. 3CH and 4CH views were defined in SAX cine images by common points in LV apex and center of mitral ring, and third point for 3CH view in aortic root and for 4CH view in lateral aspect of right ventricular free wall. Respective 3CH and 4CH cine images were acquired.

Imaging planes to acquire images oriented along LA long axis were defined by anatomical landmarks in the SAX cine images. These imaging planes were defined so that they would be analogous to LV long axis images. Most dorsal aspect of LA in the middle of pulmonary veins and center of mitral ring were two constant points. Third point was middle of atrial septum, aortic root, and LA appendage ostium to acquire LA four-chamber (LA4CH), three-chamber (LA3CH), and two-chamber (LA2CH) images, respectively.

ECG triggering for R wave was used to initiate image acquisition.

### CMR analysis

LV volume, ejection fraction and mass were measured with standard protocols by QMass MR software® (version 8.1, Medis Medical Imaging Systems, Leiden, the Netherlands). These analyzes were performed by a reader with 15 years of experience with CMR analysis (HR).

Further analyzes were performed by a reader with two years of experience with CMR analysis (JK) with freely available Medviso Segment software version 3.0 R7694 (http://segment.heiberg.se) [19]. All parameters are summarized in Additional file 1: Table 3.

**Fig. 1:**
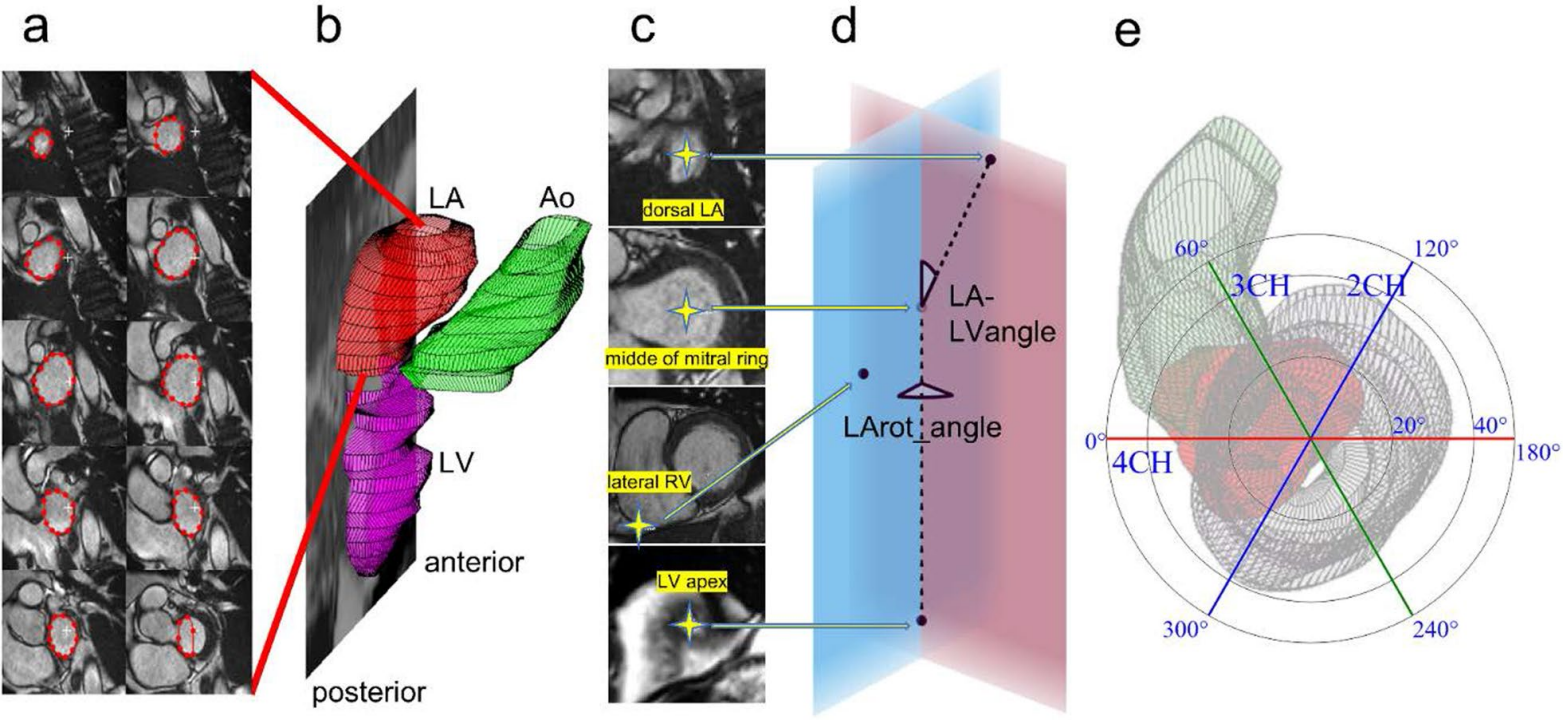
**a** Segmentation of left atrium (LA) in short axis (SAX) cine images at LA maximum volume. **b** LA (red) and left ventricle (LV, purple) shown in LV end-systolic phase according to the segmentation. 3D segmented LA maximum volume is 91.4 mL. Ascending aorta (Ao, green) is segmented roughly to aid in visualization. Displayed long axis plane is generated automatically by software from SAX images and represents roughly 2-chamber plane. **c** 3D coordinates for dorsal LA, middle of mitral ring, lateral right ventricle, and LV apex. **d** Illustration of 3D coordinates being used to define LA and LV long axes, and to calculate both LA long axis deviation angle from LV long axis (LA-LVangle) and LA long axis rotation angle in relation to 4CH plane (LArot_angle). Blue plane represents 4CH plane. A plane in which both LV and LA long axis lie is shown in red. **e** Data is later visualized in a polar graph where both LA axis deviation angle and LA axis rotation angle are shown simultaneously. Wireframe models of segmented LV (only wireframe), LA (red) and ascending aorta (green) are displayed in the background from LV apex perspective. *2CH* two-chamber, *3CH* three-chamber and *4CH* four-chamber

3D segmented LA volume (LAV_SAX_) was measured from short axis cine images (Fig. 1a, b). The endocardial border was manually drawn on each slice containing parts of the LA, and the software automatically produced the implicated volume by the sum of volumes defined in each slice. Care was taken to appropriately detect LA near the atrioventricular border. Partial volume effect is most apparent at the LA roof. Adjacent image slices both anatomically and temporally were inspected to aid manual segmentation in these areas. The LA appendage and pulmonary veins were excluded. Time frame of LA maximum volume was identified visually by browsing through LA SAX images close to LV end-systole, in contrast to selecting the time frame at the minimum LV volume, as we noted that LA maximum volume occurred typically slightly later in the cardiac cycle than the LV minimum volume. LA minimum volume was the volume at the first time frame of the cycle. LAV_SAX_ was considered the true LA volume. LA cyclic volume change was calculated by maximum LAV_SAX_ – minimum LAV_SAX_, and LA expansion index as LA cyclic volume change divided by minimum LAV_SAX_ to describe LA volume dynamics.

**Fig. 2:**
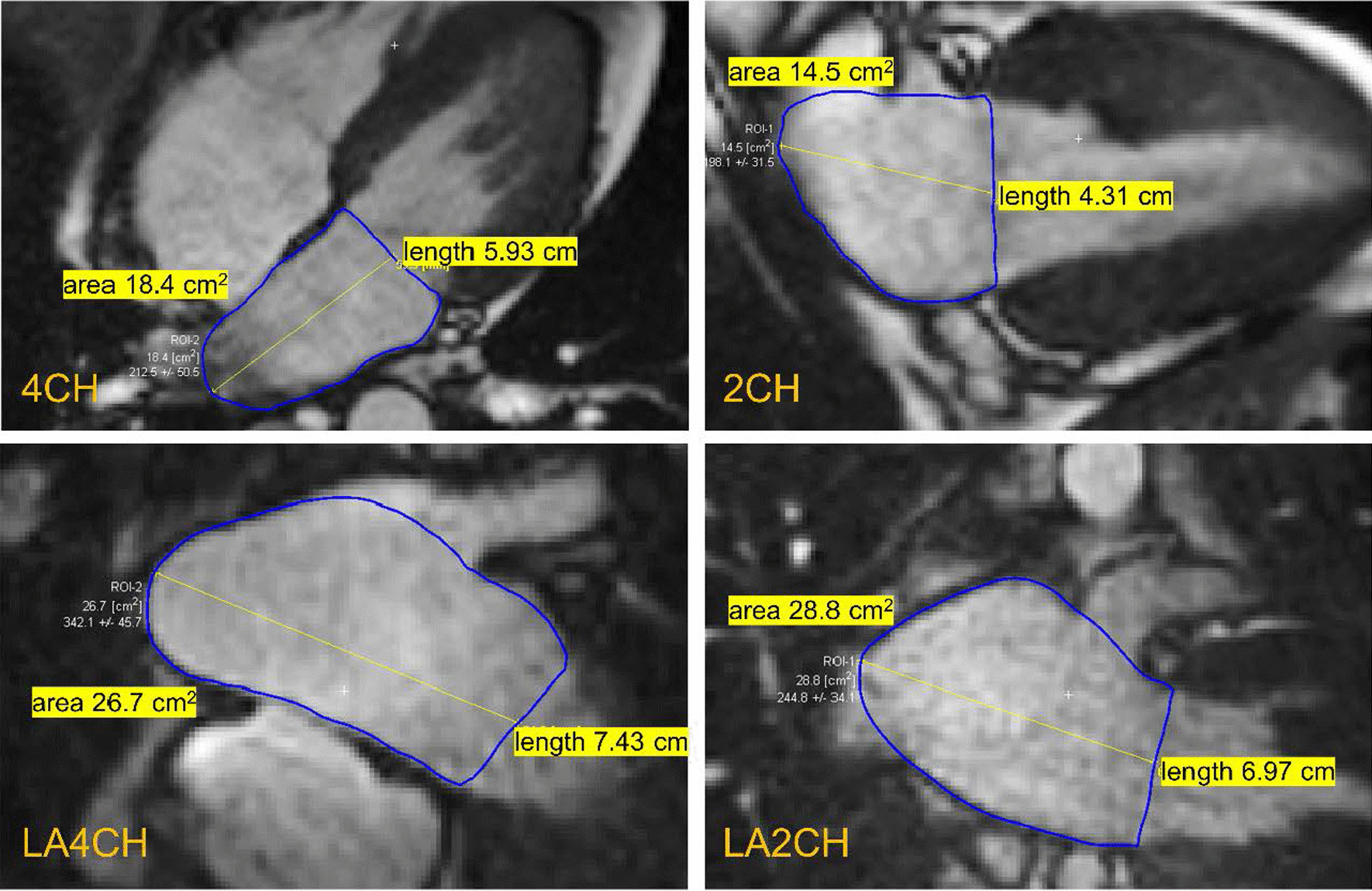
Left atrial cross-sectional largest areas and long axis lengths for left atrial volume calculation by area-length method. Left ventricular (LV) long axis-oriented left atrial (LA) images (top row) and LA long axis-oriented images (bottom row) at maximum LA volume. Calculated LA volumes are 52.6 mL (error − 38.8 mL) with LV oriented images and 93.8 mL (error + 2.4 ml) with LA oriented images. Same study subject as in Fig. 1. Planimetry area in LA oriented 2CH image is roughly twice the area in LV oriented 2CH view. *2CH* two-chamber, *4CH* four-chamber, *LA2CH* left atrium oriented two-chamber, *LA4CH* left atrium oriented four-chamber

We calculated the LA volumes by biplane area-length method both from LV long axis-oriented cine images (LAV_AL-LV_) and LA axis-oriented images (LAV_AL-LA_) also in two cardiac phases. Here, LA cross-sectional areas and the corresponding LA lengths were defined in 2CH and 4CH images for the conventional method, and in LA oriented LA2CH and LA4CH images for the novel method (Fig. 2). The volumes were calculated by the biplane area-length method equation $\frac{8}{3\pi } \times \frac{A1 \times A2}{L}$, where A1 and A2 are the corresponding planimetry areas and L is the shorter of the two measured LA axis lengths. Calculated LA volume errors were defined by subtracting LAV_SAX_ from calculated LA volumes.

The LA volumes were indexed to body surface area to assess volume abnormalities, but non-indexed volumes were used otherwise, as we aimed to assess the performance of these different methods to detect the correct chamber volume. LA maximum volume abnormality was defined by LAV_SAX_ ≥ 54 mL/m^2^ in women and LAV_SAX_ ≥ 53 mL/m^2^ in men [9] and for LAV_AL-LV_ ≥ 55 mL/m^2^ for both sexes [20]. There are no available upper normal limit data for LAV_AL-LA_ as it is introduced in this study as a novel method.

3D LV and LA long axes, and the 4-chamber planes were then determined. From the SAX cine images four points were defined, and their 3D coordinates exported to numerical computation software (GNU Octave version 4.4.0) for 3D angle measurements*.* These points included the center of the most dorsal part of the LA, LV apex, and center of the mitral ring both in end-diastolic and end-systolic time frame. The most lateral point of the right ventricular free wall at the level of mid right ventricular cavity was defined in LV end-diastole (Fig. 1c). LA axis was defined as the line connecting dorsal LA and center of mitral ring, and LV axis as the line connecting the center of the mitral annulus with the LV apex. Then the 3D angle between these two lines was calculated representing the LA long axis deviation from the LV long axis (LA-LVangle, 0 degrees represents parallel LA and LV long axes). To test inter-observer reproducibility for LA-LVangle measurement, 20 randomly selected study subjects were selected and analyzed by another reader with three years of experience with CMR analysis (PP).

The LA axis rotation angle to 4-chamber plane (LArot_angle) was calculated as follows: The 4-chamber plane was defined by 3D points in LV apex, center of mitral ring and RV lateral wall at LV end-diastole. A second plane was defined in SAX images by 3D points in LV apex, center of mitral ring, and dorsal LA. Both LV and LA long axes lie in this second plane. Then the angle between these two planes was calculated to represent LArot_angle (Fig. 1c–e).

**Fig. 3:**
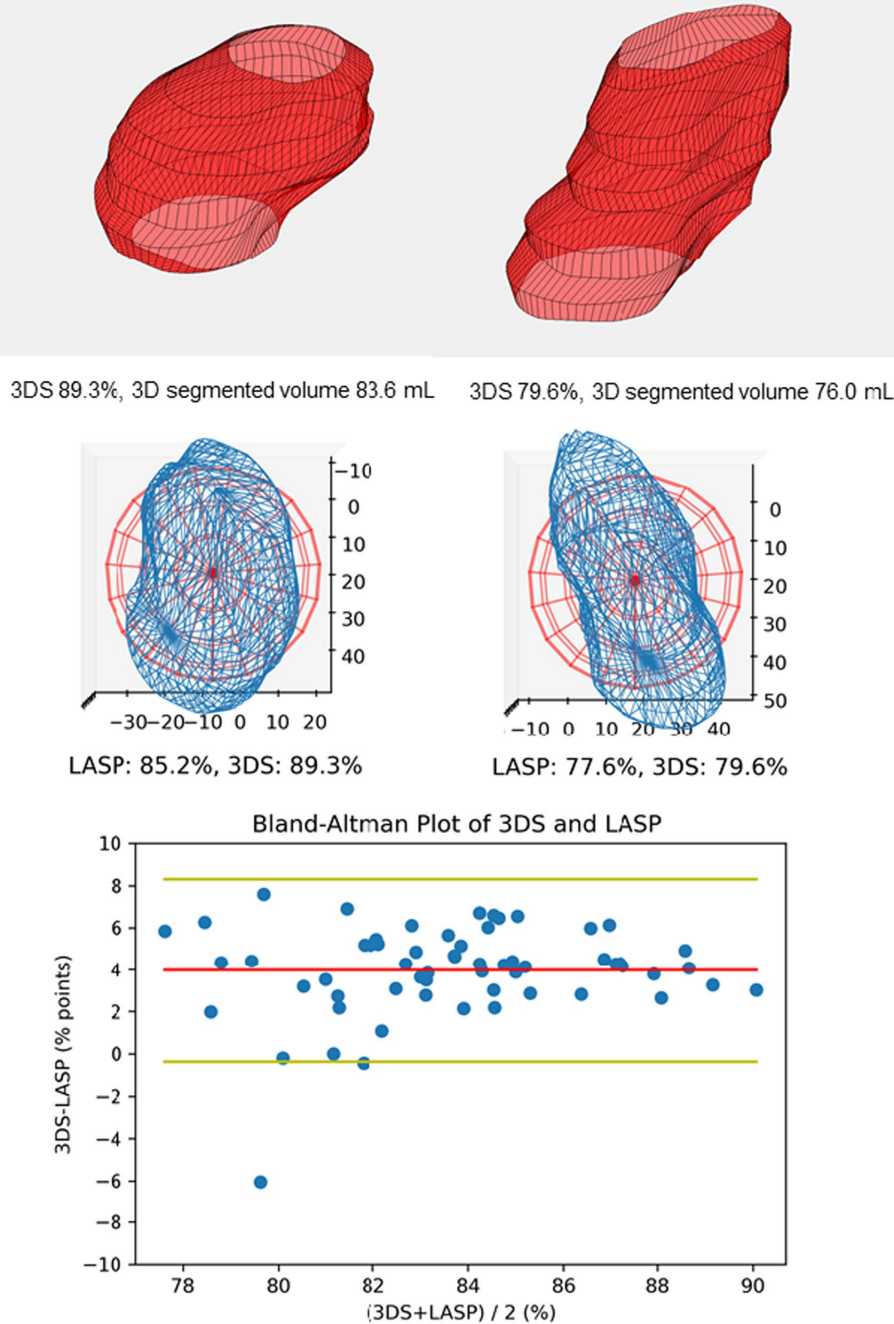
Top row—3D sphericity indices (3DS) of two cases at maximum left atrial volumes. Detected volumes and left atrial surface area was used for 3D sphericity calculation. Pulmonary vein and left atrial appendage ostia, and mitral annular openings were included to LA surface area. Middle row—Wireframe models of 3D segmented left atria in blue. Best fitted spheres are shown in red for left atrial sphericity (LASP) calculation. Study subjects are same as in top row. Bottom row—Bland–Altman plots of left atrial sphericity and 3D sphericity. Red line indicates bias, yellow lines indicate limits of agreement (bias ± 2SD). Bottom row—Bland–Altman plots of left atrial sphericity and 3D sphericity. Red line indicates bias, yellow lines indicate limits of agreement (bias ± 2SD). *3DS* 3D sphericity, *LASP* left atrial sphericity

To assess LA morphology in this study, we calculated a parameter describing LA sphericity. We defined 3D sphericity (3DS) as the ratio of surface area of a sphere with same volume as LA to actual measured left atrial surface area including mitral ring area by equation $$3$$$${\text{DS}} = \frac{{\pi^{\frac{1}{3}} \left( {6 \times {\text{LAV}}} \right)^{\frac{2}{3}} }}{{{\text{LA }}\;{\text{surface }}\;{\text{area}}}}$$ [21] (Fig. 3, top row). This approach enabled us to account all morphological details to the surface area that were included in the LA segmentation. As the same segmentation data was used for volume calculation, no additional input was needed. LA segmentation data from Segment analysis software was exported to open source 3D modeler software FreeCAD (Version 0.18, available from http://www.freecadweb.org) to measure LA surface area. Intra-observer reproducibility in 20 random subjects was tested for this novel method by repeated LA segmentation (JK) at their maximum volumes and proceeding with 3DS calculation as described above. This also yielded data to assess intra-observer LAV_SAX_ repeatability. Analysis time for these repeated segmentations was recorded in 18 subjects.

To allow comparison to our novel method of 3D sphericity, another three-dimensional index of sphericity as proposed by Bisbal et al. [16] was determined. This left atrial sphericity (LASP) was calculated by using self-coded software in Spyder integrated development environment for scientific programming in the Python language: The segmented LA model is composed of triangles defined by 3D segmentation points. Center of mass was determined first for the model. Then average radius (AR) from this center of mass was calculated as average of radii of all model triangles weighted by triangle areas. Best fitted sphere was defined as a sphere with its center at center of mass and radius of AR. Coefficient of variation of the AR weighed by triangle areas was calculated (coefficient of variation of the sphere, CVS) and from this LASP by (1-CVS) * 100% (Fig. 3, middle row). With this square root of summed squared differences is applied in calculation in contrast to our 3DS method, so lower values are expected with this method. Source code for calculating these parameters and for model visualization is available at https://github.com/JouniKuusisto/3d_sphericity.

### Statistical analysis

SPSS (IBM SPSS Statistics for Windows, Version 25.0. Armonk, NY: IBM Corp) was used for statistical analysis. Normality of continuous variables was tested using the Shapiro–Wilk test. Continuous variables are presented as mean ± standard deviation for normally distributed variables or median (interquartile range) for non-normally distributed variables.

To study agreement of calculated LA volumes to 3D segmented volumes and inter-observer and intra-observer repeatability Bland–Altman analysis [22] was performed. Intraclass correlation (ICC) coefficients were determined by using a two-way mixed model for consistency. Single measures ICC is reported with 95% confidence interval and a significance level of 0.05. Pearson correlation coefficients or Spearman’s rho were calculated according to normality of variables. Tukey’s hinges were used to define median and interquartile range.

## Results

Clinical and basic CMR characteristics of study participants appear in Table 1. Bland–Altman statistics and ICCs for all repeated measurements are presented in Table 2, whereas Bland–Altman plots appear in Additional file 1: Figures 1, 2, 3 and 4. Analysis time for repeated maximum LA volume segmentation was 3 min 45 s ± 1 min 20 s (mean ± SD).

**Table 1:** Clinical and basic cardiac magnetic resonance characteristics

	Study population (n = 60)
Women	30 (50%)
Age	43.5 (25.0–47.0)
Weight (kg)	82.8 ± 17.7
Height (cm)	173 ± 9
Body mass index (kg/m^2^)	27.5 ± 5.0
Body surface area (m^2^)	1.99 ± 0.25
Heart rate (bpm)	67.8 ± 12.3
Systolic blood pressure (mmHg)	132 ± 18
Diastolic blood pressure (mmHg)	88.5 ± 9.3
LVEDV (ml/m^2^)	84.0 (77.5–92.2)
LVESV (ml/m^2^)	30.7 ± 6.6
LVEF (%)	63.9 ± 4.9
Left ventricular mass (g/m^2^)	54.8 (48.8–60.1)

Values are expressed as n, mean ± SD or median (interquartile range). Blood pressure was measured after image acquisition

*LVEDV* left ventricular end-diastolic volume, *LVEF* left ventricular ejection fraction, *LVESV* left ventricular end-systolic volume, *SD* standard deviation

**Table 2:** Bland–Altman statistics of repeated measurements (n = 20)

	Bias (95% CI)	Limits of agreement (bias ± 2SD)	ICC (95% CI)
*Intra-observer*			
Maximum LAV_SAX_ (mL)	− 0.9 (− 2.3 to 0.5)	(− 6.9 to 5.0)	0.98 (0.96–0.99)*
3DS at maximum LA volume (% points)	− 0.4 (− 0.9 to 0.2)	(− 2.8 to 2.1)	0.94 (0.86–0.98)*
LA axis rotation angle to 4CH plane (°)	1.5 (− 0.9 to 4.0)	(− 9.0 to 12.1)	0.94 (0.85–0.97)*
*Inter-observer*			
End-diastolic LA-LV angle (°)	0.1 (− 1.8 to 2.0)	(− 8.1 to 8.3)	0.77 (0.50–0.90)*
End-systolic LA-LV angle (°)	− 0.6 (− 2.5 to 1.3)	(− 8.7 to 7.5)	0.81 (0.58–0.92)*

*3DS* 3D sphericity, *CI* confidence interval, *ICC* intraclass correlation, *LA-LV angle* 3D angle between left atrial and left ventricular axes, *LAV*_*SAX*_ measured left atrial volume by 3D segmentation, *SD* standard deviation

*p < 0.001

### Left atrial volumes

LA volumetric data is presented in Table 3. In eight study subjects indexed maximum LAV_SAX_ was increased (5 mildly, 3 moderately abnormal, all men). Indexed maximum LAV_AL-LV_ was abnormal in one male subject (no false positives, 7 false negatives compared to classification with LAV_SAX_).

**Table 3:** Left Atrial Volumes, 3D Angles, and Sphericity Indices (n = 60)

		Volume abnormal (n)
*LAV*_*SAX*_		
max (mL/m^2^)	44.6 ± 7.5	8
max (mL)	88.4 ± 18.0	
min (mL)	38.6 ± 10.7	
*LAV*_*AL-LV*_		
max (mL/m^2^)	39.7 ± 7.7	1
max (mL)	78.6 ± 17.1	
min (mL)	31.2 ± 10.0	
*LAV*_*AL-LA*_		
max (mL/m^2^)	50.1 (44.2–57.5)	NA
max (mL)	98.7 (89.4–109.9)	
min (mL)	43.4 (37.8–55.5)	
*LA and LV long axes deviation angle*		
end-systole (deg)	21.1 ± 6.4	
end-diastole (deg)	28.3 ± 6.2	
cyclic angle change (deg)	7.3 ± 4.7	
*LA axis rotation to 4CH plane*		
end-diastole (deg)	20.5 ± 18.0	
*Left atrial 3D sphericity index (3DS)*		
end-systole (%)	85.6 ± 3.2	
end-diastole (%)	81.7 (79.5–83.8)	
*Left atrial sphericity index (LASP)*		
end-systole (%)	81.7 ± 2.9	

*4CH* 4-chamber plane, *LA* left atrium, *LAV*_*AL-LV*_* and LAV*_*AL-LA*_ left atrial volume calculated by area-length method from left ventricular long axis images and left atrial long axis images, respectively, *LAV*_*SAX*_ measured left atrial volume by 3D segmentation, *LV* left ventricle, *NA* not applicable

**Fig. 4:**
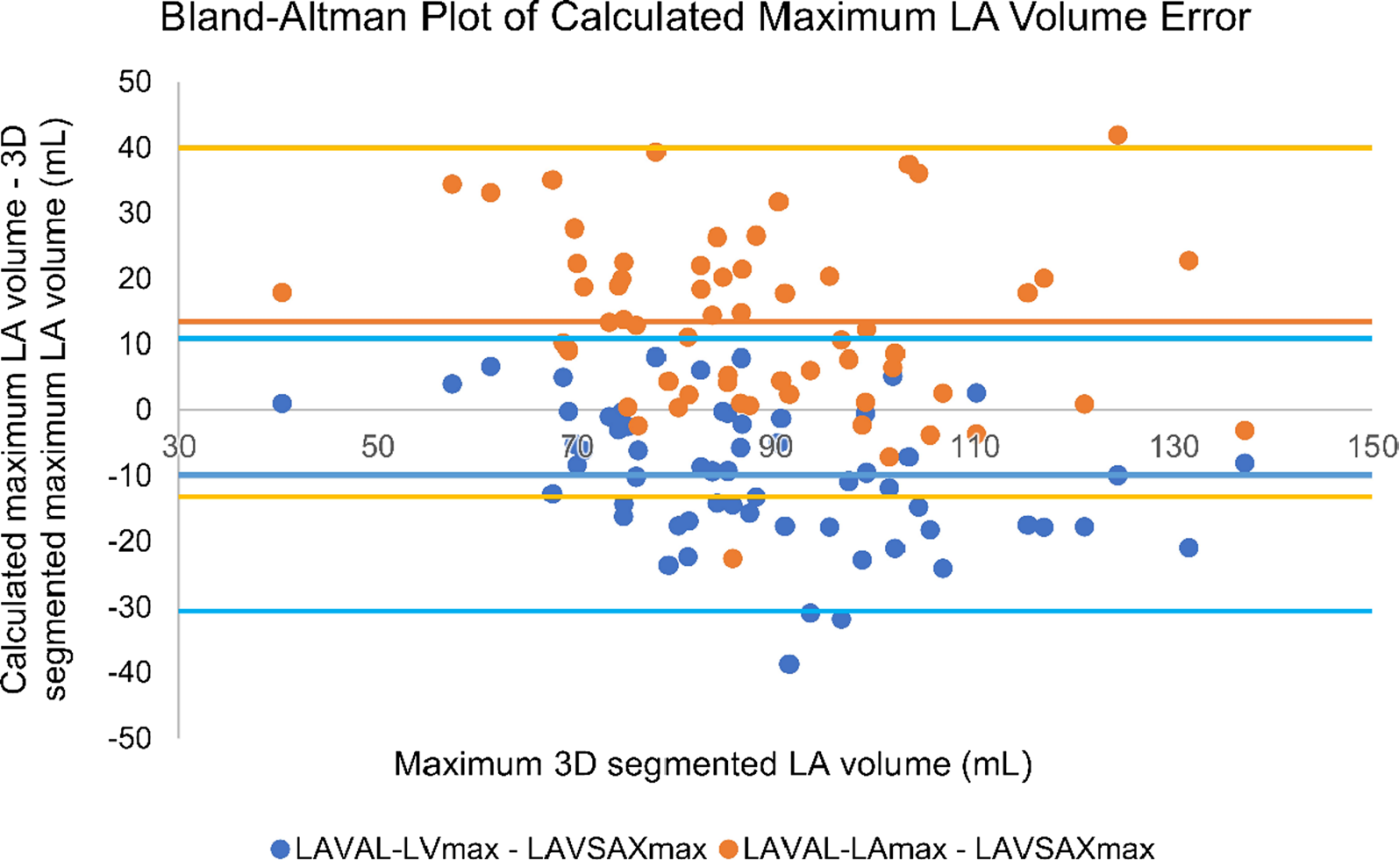
Bland–Altman plots of left atrial volumes by area-length calculation and 3D segmentation. Blue and orange dots represent calculated left atrial volumes from left ventricular and left atrial axis-oriented images, respectively. Dark blue and orange lines on right represent bias and light blue and yellow lines represent limits of agreement (mean ± 2 SD). *LA* left atrium, *LAV*_*AL-LA*_ LA volume calculated by area-length method from LA axis-oriented images, *LAV*_*AL-LV*_ LA volume calculated by area-length method from LV axis-oriented images, *LAV*_*SAX*_ LA volume by 3D segmentation

Bland–Altman statistics and ICCs of 3D segmented and calculated LA volumes are shown in Table 4 and Bland–Altman plots in Fig. 4. Additional plots are shown in Additional file 1: Figures 5 and 6. Calculated LA volume bias to 3D segmented LA volume (LAV_SAX_) was negative with conventional LV long axis (LAV_AL-LV_) and positive with LA long axis (LAV_AL-LA_) oriented images in both maximum and minimum LA volumes. LAV_AL-LV_ bias had small negative correlation to LAV_SAX_ (maximum volume R = − 0.37, *p* = 0.003, and minimum R = − 0.43, *p* = 0.001), but no volume dependency on bias was observed with LAV_AL-LA_ (R = − 0.18, *p* = 0.16 and R = 0.15, *p* = 0.91, respectively).

**Table 4:** Bland–Altman statistics of calculated left atrial volumes to 3D segmented volumes (n = 60)

	Bias (95% CI)	Limits of agreement (bias ± 2SD)	ICC (95% CI)
max LAV_AL-LV_ (mL)	− 9.9 (− 12.5 to − 7.2)	(− 30.6 to 10.9)	0.83 (0.72–0.89) *
max LAV_AL-LA_ (mL)	13.4 (10.0–16.9)	(− 13.1 to 40.0)	0.76 (0.63–0.85) *
min LAV_AL-LV_ (mL)	− 7.4 (− 9.3 to − 5.5)	(− 22.0 to 7.2)	0.75 (0.62–0.84) *
min LAV_AL-LA_ (mL)	8.8 (6.3–11.2)	(− 10.0 to 27.6)	0.75 (0.61–0.84) *

*Bias* mean of difference to 3D segmented left atrial volume, *CI* confidence interval, *ICC* intraclass correlation, *LAV*_*AL-LV*_* and LAV*_*AL-LA*_ left atrial volume calculated by area-length method from 2D images oriented along left ventricular and left atrial long axes, respectively, *SD* standard deviation

*p < 0.001

### Left atrial axis angles

LA-LV axis deviation and LA axis rotation angle data are presented in Table 3. In men the LA-LV deviation angle showed a trend for greater angle (end-diastolic (ED) angle 26.9 ± 6.2° in women and 29.8 ± 5.9° in men, *p* = 0.07 and end-systolic (ES) angle 19.5 ± 5.9 and 22.6 ± 6.4, *p* = 0.06, respectively). Cyclic LA-LV angle change was similar in both sexes (7.4 ± 3.9° in women and 7.2 ± 5.5° in men, *p* = 0.83). BSA and weight had a small but statistically significant positive correlation to ED LV-LA angle, (BSA R = 0.260, *p* = 0.045 and weight R = 0.255, *p* = 0.049). Age, body mass index, height, LV ejection fraction, or indexed LV mass did not have statistically significant correlation to any angles. Small but statistically significant negative correlation was observed with cyclic LA-LV angle change and indexed LV end diastolic volume (R = − 0.259, *p* = 0.046). LA axis rotation angle did not differ between sexes or correlate with any other baseline data. None of the study subjects had chest wall or thoracic spine abnormalities and all had normal thoracic aortas.

### Calculated left atrial volume error and left atrial axis angles

**Fig. 5:**
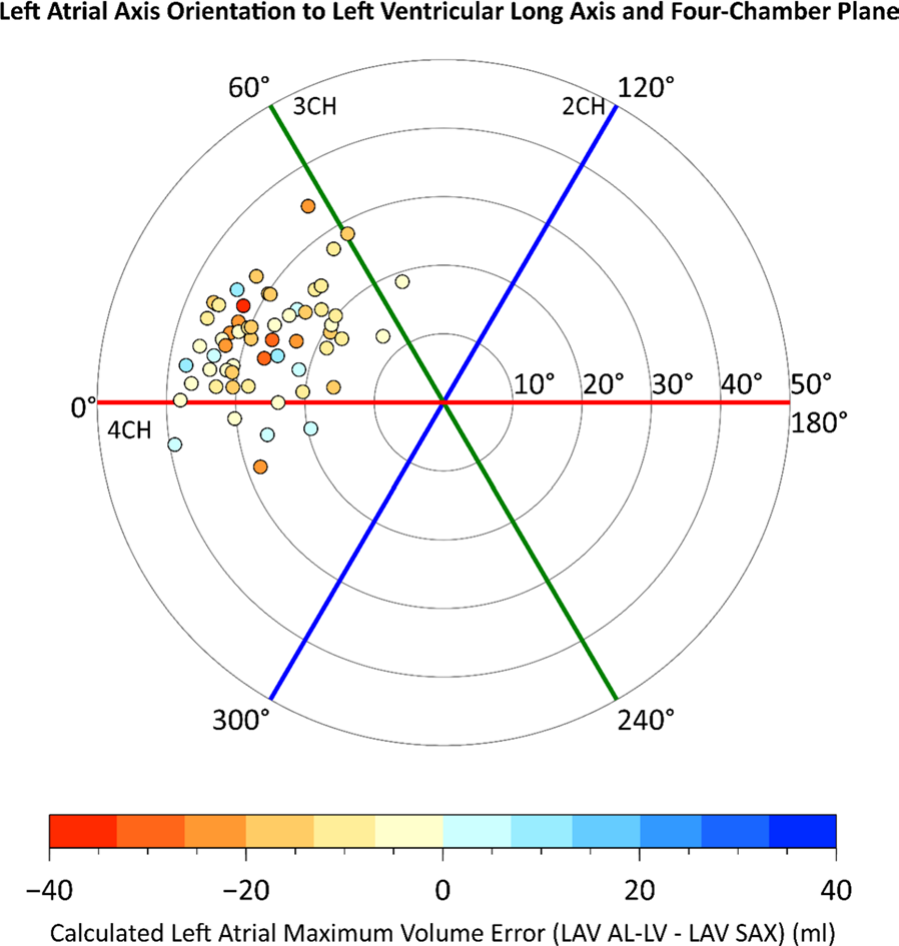
Polar plot of left atrial (LA) axis orientation and calculated LA volume error. LA and left ventricular long axes deviation angles are shown as radial coordinates. LA axis rotation angles are shown in angular coordinates. Both angles are represented at end-diastolic phase. Data point color indicates error of calculated LA volume. Red line represents 4-chamber plane, green line 3-chamber plane, and blue line 2-chamber plane. *LAV AL-LV* LA volume calculated by area-length method from LV axis-oriented images, *LAV SAX* LA volume by 3D segmentation

Errors of calculated LA volumes were not associated with the angles between LA and LV axes, or LA axis rotation to the 4-chamber plane. LA-LV axis deviation and LA rotational angle data with calculated volume errors are presented in Fig. 5. Scatter plots for calculated volume error and LA-LV axis deviation appear in Additional file 1: Figure 7.

### Left atrial volume dynamics and left atrial and ventricular long axis angles

LA cyclic volume change or LA expansion index did not have significant correlation to any LA-LV angle parameters. Maximum indexed LAV_SAX_ had small negative correlation to LA-LV ED angle and both maximum and minimum indexed LAV_SAX_ to cyclic LA-LV angle change (R = − 0.353, *p* = 0.006; R = − 0.356, *p* = 0.005; and R = − 0.261, *p* = 0.044, respectively).

### Left atrial sphericity

Left atrial sphericity data are presented in Table 3. 3DS and LASP had an ICC of 0.750 (95% CI 0.614–0.843). Bland–Altman plot is shown in bottom row of Fig. 3. 3DS or LASP did not correlate with LA absolute or indexed volumes or LA volume dynamics. LAV_AL-LV_ error was not correlated to either sphericity indices. With 3DS a small negative correlation with LAV_LA-LA_ error was observed (at maximum LA volume R = − 0.376, *p* = 0.003 and at minimum LA volume R = − 0.264, *p* = 0.04, Additional file 1: Figures 8 and 9). Small negative correlations were observed with 3DS and LA-LV angles (end-diastolic angle R = − 0.255, *p* = 0.049, end-systolic-angle R = − 0.393, *p* = 0.002). Same associations were observed with LASP at maximum LA volume: correlation coefficients for maximum and minimum LAV_AL-LA_ errors, and end-diastolic and end-systolic LA-LV angles were − 0.454 (*p* < 0.001), − 0.535 (*p* < 0.0001), − 0.295 (*p* = 0.22), and − 0.427 (*p* = 0.001), respectively.

## Discussion

In this study we examined agreement of different methods of determining LA size with CMR in a study population with no apparent heart disease. As a novel method, we calculated volumes from specific LA long axis-oriented images by area-length method to study whether this would increase accuracy of volume detection over calculations based on conventional LV long axis-oriented images. Manual 3D segmentation served as a gold standard for volume measurement. We investigated the role of differing LA and LV long axes, rotation of LA axis to conventional LV imaging planes, and LA sphericity in introducing error to LA volume detection by these calculation methods. To the best of our knowledge, 3D orientation of the LA axis and its cyclic variation has not been reported before. We used 3D segmentation data, as it provides the complete shape of the LA in addition to its volume, to calculate 3D sphericity index as a novel method to assess LA sphericity in addition to left atrial sphericity proposed by Bispal et al. [16] Short axis images for LA 3D volume measurement were selected for clinical feasibility, so that both LA and LV 3D volumes can be measured from a single cine series.

LA volume detection was not more reliable when we calculated LA volume from LA oriented images, in comparison to the conventional area-length method. Ranges of limits of agreement were similar in addition to similar sized but opposing biases with both methods. We anticipated that LV-oriented LA images would more likely have foreshortening and smaller cross-sectional areas, and thus result in negative bias in calculated LA volumes, which was confirmed. The LAV_AL-LV_ method appeared prone to false negatives, compared to the LAV_SAX_ method as a gold standard. With our proposed LAV_AL-LA_ we seemed to introduce error which was dependent on left atrial sphericity. Conventional 2D method for maximum LA volume calculation showed a negative absolute bias of − 9.9 mL. We also observed quite large discrepancy in classification as one study subject was classified to have abnormal volume with LAV_AL-LV_ in contrast to 8 subjects with 3D segmentation. Normal ranges with 3D segmentation do however have less established thresholds for classification. Expected number of abnormal volume in a population of 60 study subjects would lie in between these numbers. In Bland–Altman analysis we observe some cases with quite a large discrepancy in volumes. True misclassification with 2D method likely exists, which can have an impact on clinical practice.

Both sphericity indices 3DS and LASP correlated inversely to LA-LV angles and error of LAV_AL-LA_. The left atria with less-spherical shape tend to lie in greater angle to LV axis in this study population. We speculate that anatomical relation of LA to aortic root might be a factor contributing to this finding. We did not observe a correlation of error of LAV_AL-LA_ to LA-LV-angle. It seems likely that less-spherical LA shape introduces error to this 2D method, which is unrelated to greater LA-LV angle, even though sphericity is correlated to both. Conventional 2D method may omit outlying areas of these non-spherical forms and be less susceptible to overestimation of volume. The role of sphericity in the error of calculated volumes can be considered low with LAV_AL-LV_. It might be so that in this study population without known structural heart disease the association of LA-LV angles and LA sphericity might be due to normal variation of anatomy and that in atrial dilatation LA-LV angle or LA sphericity might contribute more to calculated volume error—a study in a population with significant heart disease enlarging the atrium might give more insight to this.

We used 3D sphericity indices, which are devoid of geometrical assumptions or rater discretion after LA 3D segmentation. Although these measures can indicate deviation of the LA morphology from a perfect sphere, they do not specify how this deviation from sphereness is distributed. In atrial fibrillation, the freedom of arrhytmia recurrence after catheter ablation has been suggested to be associated with asymmetrical left atrial dilatation and regional left atrial wall deformity [23, 24]. Regional deformities might further contribute to error in volume calculation by 2D methods. This was however not studied in the scope of this study.

LA axis mean rotation to 4-chamber plane of 20.5° translates to LA axis lying roughly between four- and three-chamber planes and almost perpendicular to the two-chamber plane. This leads us to question the rationale of using two-chamber view for atrial volume calculation and rather urges including three-chamber view to LA volume analysis instead. However, this conclusion is based on imaging planes lying in approximately 60 degrees angle to one another, which might not be the case in the clinical setting. Inclusion of the three-chamber plane was not investigated in this study. Furthermore, left atrial axis angles were not associated to observed error of calculated LA volume in our data. Mean cyclic angle change of LA and LV long axes was 7.3°. At end-systole the axes were more aligned. During LV systole the mitral annulus moves towards LV apex and away from dorsal LA, but LV apex and dorsal LA points do not move significantly in the thoracic cavity. This action shortens LV and elongates LA long axis. The change of LV long axis length is greater than LA long axis, as the angle is smaller at end-systole. This angular change could have an effect on blood flow from pulmonary veins to LA or through the mitral annulus but was not investigated in this study. LA-LV angle was calculated to assess its contribution in volume calculation error. This measure could also be used for studying cardiac structure relations in cardiac pathology, i.e. in LA or aortic root dilatation. This study population gives insight on repeatability in normal cardiac anatomy. In pathology some methodological challenges could emerge: enlargement of dorsal area of LA could obscure selection of mid-point in dorsal area [25], or that span of LA-LV angles might be narrower in LA dilatation lessening its diagnostic potential.

We approximated that six extra SAX slices to cover LA would add roughly 3 min of extra time during imaging and another 3–5 min of manual segmentation of the LA in one cardiac phase. Measured mean time of repeated LA segmentation was indeed a little < 4 min. Direct volume measurement over a whole cardiac cycle enables assessment of LA dynamics (time volume curve) more closely and may provide added information on cardiac conditions. With manual segmentation this is not currently feasible in the clinical setting. However, machine learning has been demonstrated to be comparable to human inter-observer variability in LV and right ventricle segmentation, determining LA areas in long axis images, and providing segmentation of the entire cardiac cycle automatically [26]. These advancements will most likely help overcome these obstacles for direct volume measurements in the future and will likely replace area-based 2D methods for volume determination in long term both in research and clinical applications.

In future studies the inclusion of 3-chamber view for 2D volume calculation could be investigated with both 2D methods. LAV_AL-LA_ method did not show volume dependent error and its overestimation could be addressed by adjusting the calculation formula with larger dataset. The limits of agreement however remain quite large in both 2D methods. Clinical utility of conventional 2D method still remains as reference values are more established.

## Limitations

The study population consisted of subjects with no known heart disease. Some aspects of correlations or causalities might go unnoticed when variation is confined to normal ranges, rather than that of pathology. Therefore, these results cannot be directly extrapolated to patients with enlarged left atria. 3D segmentation, which was considered the true volume in our study, is not devoid of errors either. These include partial volume effect, which can be exaggerated with greater LA axis deviation from LV axis, as SAX images are acquired along LV axis; and basis area segmentation where LA, mitral apparatus and LV coincide. The potential added accuracy of 2D volume calculation by inclusion of the 3-chamber view was not investigated.

## Conclusions

We demonstrated that in this study population the error in LA volume calculation by the area-length method is more dependent on the geometrical assumptions of LA structure, rather than orientation of LA axis or LA sphericity. The error in calculated volumes cannot be mitigated by using LA specific long axis images for volume calculation or by analyzing known sources of error.

## Supplementary Information

**Additional file 1:** Supporting Information.

## Data Availability

The datasets used and/or analyzed during the current study are available from the corresponding author on reasonable request.

## Abbreviations

2CH: Two-chamber plane
2D: Two-dimensional
3CH: Three-chamber plane
3D: Three-dimensional
3DS: 3D sphericity
4CH: Four-chamber plane
CI: Confidence interval
CMR: Cardiovascular magnetic resonance imaging
ECG: Electrocardiogram
ED: End-diastolic
ES: End-systolic
ICC: Intraclass correlation
LA: Left atrium
LA2CH: Left atrial two-chamber plane
LA3CH: Left atrial three-chamber plane
LA4CH: Left atrial four-chamber plane
LA-LVangle: 3D angle between left atrial and left ventricular long axes
LArot_angle: Left atrial long axis rotation angle to four-chamber plane
LASP: Left atrial sphericity
LAVAL-LA: Left atrial volume calculated by area-length method from 2D images oriented along left atrial long axis
LAVAL-LV: Left atrial volume calculated by area-length method from 2D images oriented along left ventricular long axis
LAVSAX: Measured left atrial volume by 3D segmentation
LV: Left ventricle
LVEDV: Left ventricular end-diastolic volume
LVEF: Left ventricular ejection fraction
LVESV: Left ventricular end-systolic volume
SAX: Short-axis
SD: Standard deviation
