# Supplementary material for: Revisiting left atrial volumetry by magnetic resonance imaging: the role of atrial shape and 3D angle between left ventricular and left atrial axis
Source: BMC Med Imaging. 2021 Nov 9;21:167. doi: 10.1186/s12880-021-00701-5 (PMC8579555; doi:10.1186/s12880-021-00701-5)
Supplement: Supplementary file 1 — Additional file 1: Supporting Information. [file 12880_2021_701_MOESM1_ESM.docx]

| \| Table 1.  Baseline characteristics of patients with cryptogenic stroke and stroke-free controls. \| \| \| \| \| --- \| --- \| --- \| --- \| \|  \| **Cases (n=30)** \| **Controls (n=30)** \| ***P*-value** \| \| **Demographics and body measurements** \|  \|  \|  \| \| Age at stroke onset or at inclusion, years \| 41 (35-44) \| 42 (35-45) \| 0.131 \| \| Gender, female \| 15 (50) \| 15 (50) \| 1.000 \| \| Height, cm \| 175 ± 9 \| 171 ± 9 \| 0.013 \| \| Weight, kg \| 87 ± 18 \| 79 ± 17 \| 0.042 \| \| Body surface area, m2 \| 2.04 ± 0.24 \| 1.93 ± 0.24 \| 0.022 \| \| Body mass index, kg/m2 \| 28.4 ± 5.0 \| 26.9 ± 5.2 \| 0.294 \|   Cardiac magnetic resonance of young adults with cryptogenic stroke and stroke-free controls: Basic structures and function. | | | |
| --- | --- | --- | --- | --- | --- | --- | --- | --- | --- | --- | --- | --- | --- | --- | --- | --- | --- | --- | --- | --- | --- | --- | --- | --- | --- | --- | --- | --- | --- | --- | --- | --- | --- | --- | --- | --- | --- | --- | --- |
|  | **Cases (n=30)** | **Controls (n=30)** | ***P*-value** |
| LV end-diastolic volume index, ml/m^2^ | 82.5 (77.9-89.3) | 84.5 (76.0-93.5) | 0.910 |
| LV end-systolic volume index, ml/m^2^ | 30.5 ± 6.0 | 31.1 ± 7.2 | 0.734 |
| LV stroke volume index, ml/m^2^ | 54.0 ± 7.0 | 55.0 ± 10.3 | 0.666 |
| LVEF, % | 64.1 ± 4.7 | 63.8 ± 5.1 | 0.820 |
| LA volume max index, ml/m^2^ | 44.5 ± 6.7 | 44.7 ± 8.3 | 0.907 |

Figure 1 Bland-Altman plot of intra-observer repeatability of maximum left atrial volume detection by left atrial 3D segmentation. Red line – bias, orange lines – limits of agreement (bias±2SD).

Figure 2 Bland-Altman plot of intra-observer repeatability of maximum 3D sphericity index. Red line – bias, orange lines – limits of agreement (bias±2SD).

Figure 3 Bland-Altman plot of inter-observer repeatability of end-diastolic angle between left atrial and left ventricular long axes. Red line – bias, orange lines – limits of agreement (bias±2SD).

Figure 4 Bland-Altman plot of inter-observer repeatability of end-systolic angle between left atrial and left ventricular long axes. Red line – bias, orange lines – limits of agreement (bias±2SD).

Figure 5 Calculated maximum LA volumes in comparison to 3D segmented LA volumes as scatter plots. Blue and orange dots represent calculated LA volumes from LV and LA axis-oriented images, respectively.

Figure 6 Left atrial volumes by area-length calculation and 3D segmentation. Calculated minimum LA volumes in comparison to true LA volumes as scatter (A) and Bland-Altman plots (B). Blue and orange dots represent calculated LA volumes from LV and LA axis-oriented images, respectively. Dark blue and orange lines on right represent bias and light blue and yellow lines represent limits of agreement (mean±2 SD).


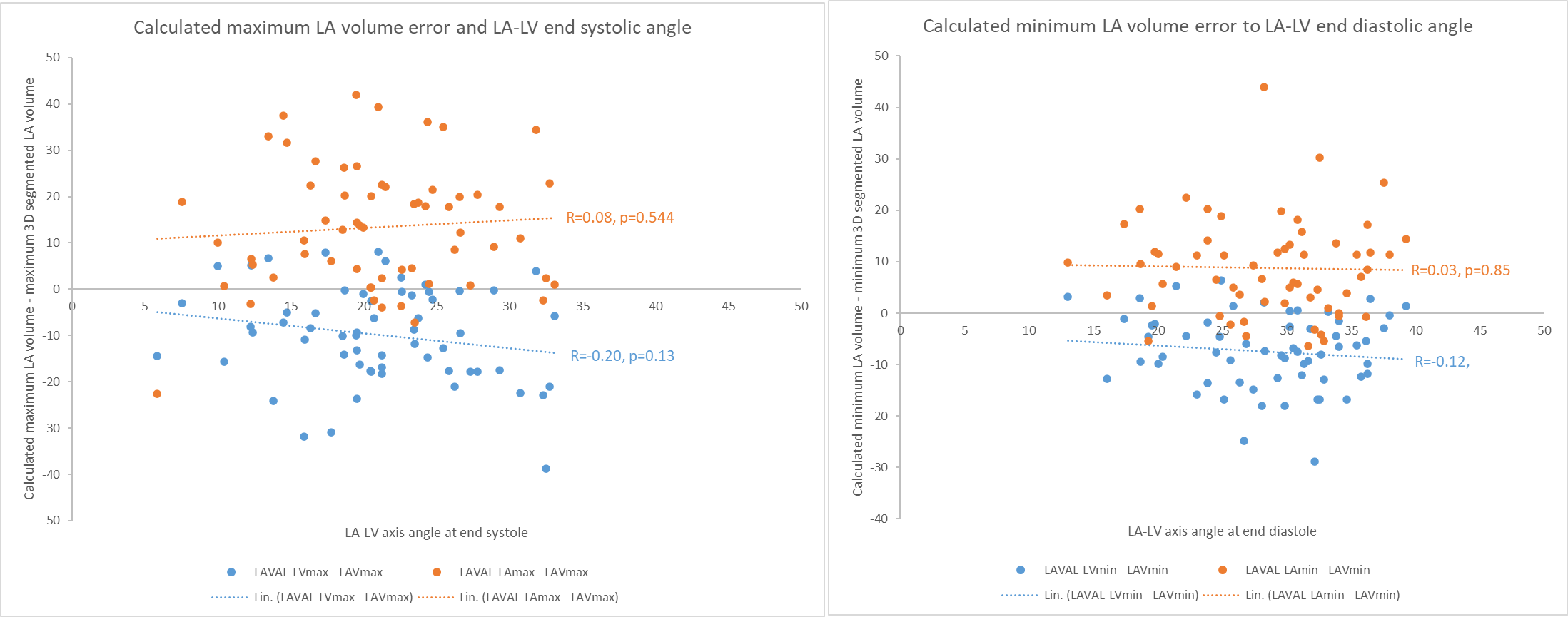


Figure 7 Relation of calculated LA volume error to angle between LA and LV long axes. Blue dots represent error of calculated LA volumes from LV oriented images and orange dots represent respective error from LA oriented images.

Figure 8 Relation of left atrial 3D sphericity and error of left atrial area-length calculated volume to 3D segmented volume at left atrial maximum volume. 3DSI – 3D sphericity index, LAVAL-LV and LAVAL-LA – left atrial volume calculated by area-length method from images along left ventricular long axes and left atrial long axes, respectively.

Figure 9 Relation of left atrial 3D sphericity and error of left atrial area-length calculated volume to 3D segmented volume at left atrial minimum volume. 3DSI – 3D sphericity index, LAVAL-LV and LAVAL-LA – left atrial volume calculated by area-length method from images along left ventricular long axes and left atrial long axes, respectively.

| **Table 2.** Cardiac magnetic resonance protocol sequence parameters | | | | | | | | | | | | | | | | | | |
| --- | --- | --- | --- | --- | --- | --- | --- | --- | --- | --- | --- | --- | --- | --- | --- | --- | --- | --- |
| **Sequence** | **Acquisition** | **ECG** | **RESP** | **Slice thickness (mm)** | **FOV read (mm)** | **FOV phase (%)** | **Base resolution** | **Phase resolution (%)** | **PE steps** | **Flip angle** | **PE direction** | **TR (ms)** | **TE (ms)** | **Averages** | **Slice spacing (mm)** | **ETL** | **Bandwidth (Hz/px)** | **Other** |
| 3-direction localizer | 2D | prosp | BH | 8 | 400 | 100 | 240 | 66 | 206 | 64 | ROW | 301,3 | 1,1 | 1 | 11,2 | 1 | 1155 |  |
| HASTE, TRA | 2D | prosp | BH | 8 | 370 | 75 | 256 | 75 | 85 | 160 | ROW | 612 | 39 | 1 | 8 | 56 | 780 |  |
| Cine, cartesian acquisition (2CH, 3CH, 4CH, LV and LA SAX) | 2D | retro | BH | 6 | 320 | 81 | 192 | 80 | 220 | 55 | ROW | 33,7 | 1,2 | 1 | 0 | 1 | 930 | 40 cine phases |
| Cine, radial acquisition (LA2CH, LA3CH, LA4CH) | 2D | retro | BH | 6 | 250 | 100 | 128 | 100 | 91 | 55 | COL | 38,1 | 1,4 | 1 | 0 | 1 | 975 | 30 cine phases |
| BH, breath hold; ECG, electrocardiogram; ETL, echo train length; FOV, field of view; HASTE, Half-Fourier-acquisition single-shot Turbo spin echo; LA, left atrium; LA2CH, left atrium oriented two chamber; LA3CH, left atrium oriented three chamber; LA4CH, left atrium oriented four chamber; LV, left ventricular; PE, phase encoding; prosp, prospective; RESP, respiration; retro, retrospective; SAX, short-axis; TE, echo time; TR, repetition time; TRA, transaxial; 2CH, two chamber; 3CH, three chamber; 4CH, four chamber. | | | | | | | | | | | | | | | | | | |

| **Table 3: CMR analysis parameters** | | | |
| --- | --- | --- | --- |
| **parameter** | **explanation** | **unit** | **images used for analysis** |
| 3DS | left atrial 3D sphericity | % | SAX |
| LA-LVangle | left atrial and left ventricular long axis deviation 3D angle | ° | SAX |
| LArot_angle | left atrial axis rotational 3D angle to 4CH plane | ° | SAX |
| LAV_AL-LA_ | left atrial volume calculated by area-length method | mL | LA long axis 2CH and 4CH |
| LAV_AL-LV_ | left atrial volume calculated by area-length method | mL and mL/m^2^ | LV long axis 2CH and 4CH |
| LAV_SAX_ | left atrial volume measured by 3D segmentation | mL and mL/m^2^ | SAX |
| LV ejection fraction | left ventricular ejection fraction | % | SAX |
| LV mass | left ventricular mass | g/m2 | SAX |
| LV volume | left ventricular volume, end-diastolic and end-systolic | mL/m^2^ | SAX |
| SAX - short axis, 2CH - two-chamber, 4CH-four-chamber | |  |  |
